# Serotonin stimulates *Echinococcus multilocularis* larval development

**DOI:** 10.1186/s13071-020-04533-0

**Published:** 2021-01-06

**Authors:** Michaela Herz, Klaus Brehm

**Affiliations:** Institut für Hygiene und Mikrobiologie, Universität Würzburg, Josef-Schneider-Straße 2/E1, 97080 Würzburg, Germany

**Keywords:** Cestode, Proliferation, Serotonin, Serotonin transporter, Tryptophan hydroxylase, Nervous system

## Abstract

**Background:**

Serotonin is a phylogenetically ancient molecule that is widely distributed in most metazoans, including flatworms. In addition to its role as a neurotransmitter, serotonin acts as a morphogen and regulates developmental processes. Although several studies have focused on the serotonergic nervous system in parasitic flatworms, little is known on the role of serotonin in flatworm development.

**Methods:**

To study the effects of serotonin on proliferation and development of the cestode *Echinococcus multilocularis*, we cloned the genes encoding the *E. multilocularis* serotonin transporter (SERT) and tryptophan hydroxylase (TPH), analyzed gene expression by transcriptome analysis and whole mount* in situ* hybridization (WMISH) and performed cell culture experiments.

**Results:**

We first characterized orthologues encoding the SERT and TPH, the rate-limiting enzyme in serotonin biosynthesis. WMISH and transcriptomic analyses indicated that the genes for both SERT and TPH are expressed in the parasite nervous system. Long-term treatment of parasite stem cell cultures with serotonin stimulated development towards the parasite metacestode stage. Mature metacestode vesicles treated with serotonin showed increased rates of incorporation of the thymidine analogue 5-ethynyl-2′-deoxyuridine (EdU), indicating stimulated cell proliferation. In contrast, treatment with the selective serotonin reuptake inhibitor paroxetine strongly affected the viability of parasite cells. Paroxetine also caused structural damage in metacestode vesicles, suggesting that serotonin transport is crucial for the integrity of parasite vesicles.

**Conclusions:**

Our results indicate that serotonin plays an important role in *E. multilocularis* development and proliferation, providing evidence that the *E. multilocularis* SERT and TPH are expressed in the nervous system of the protoscolex. Our results further suggest that the *E. multilocularis* SERT has a secondary role outside the nervous system that is essential for parasite integrity and survival. Since serotonin stimulated *E. multilocularis* metacestode development and proliferation, serotonin might also contribute to the formation and growth of the parasite in the liver.
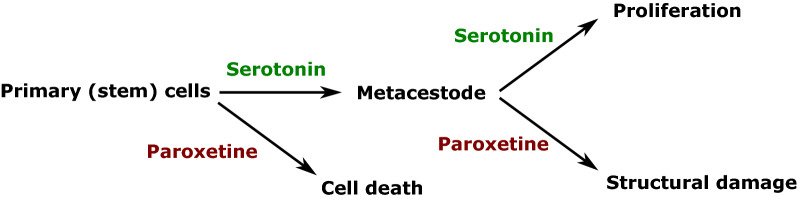

## Background

The metacestode stage of the cestode *Echinococcus multilocularis* is the causative agent of alveolar echinococcosis, a severe zoonosis prevalent in the northern hemisphere [1]. Oral uptake of infectious eggs, which harbor the oncosphere, leads to infection of intermediate hosts, such as small rodents and humans. The oncosphere hatches from the egg in the small intestine, penetrates the epithelium and gains access to the inner organs, especially the liver, where it transforms into the metacestode larval stage. The metacestode invasively grows inside the liver as a multi-vesicular parasite tissue [2]. In advanced infections of natural intermediate hosts, the metacestode vesicles form brood capsules with protoscoleces, which eventually develop into the adult stage when transmitted to a definite host or “re-differentiate” within the intermediate host towards the metacestode stage [3]. During these developmental transitions the nervous system undergoes remarkable changes [4, 5].

The morphology of the serotonergic nervous system has been described for several cestodes and trematodes [6–12], including *Echinococcus granulosus* [4, 13, 14] and *E. multilocularis* [5]. Functional studies have shown that serotonin is required for the maintenance of contractility in muscle fibers isolated from *Schistosoma mansoni* adults [15], increases motility in *S. mansoni* sporocysts [16], influences scolex and strobila motility in *Hymenolepis diminuta* [17] and increases motility of *E. granulosus* protoscoleces [4] and *Mesocestoides corti* tetrathyridia [18].

In addition to its role as a neurotransmitter, serotonin is also known to act as a morphogenic factor that influences developmental processes [19]. Depending on the context, serotonin can induce cell proliferation, differentiation or apoptosis [20–26]. Serotonin also plays an important role during traumatic regeneration of the planarian *Polycelis tenuis* [27] and influences miracidial transformation of *S. mansoni* (transition from free-living to parasitic stage) [28, 29]. However, little is known about the developmental role of serotonin in cestodes. In a previous study we and our colleagues showed that exogenously supplied serotonin induces re-differentiation of *E. granulosus* protoscoleces towards the metacestode stage [4]. Whether this is also the case for *E. multilocularis* has not been investigated to date. Furthermore, the influence of serotonin on other larval transitions, such as the development of the oncosphere towards the metacestode or the proliferation of metacestode vesicles, has not yet been addressed.

The developmental transitions of *E. multilocularis* larvae within the intermediate host can be mimicked* in vitro* using culture systems for metacestode vesicles and parasite stem cells that we have previously developed [30–32]. In the study reported here, we investigated the effect of serotonin on *E. multilocularis* proliferation and larval development. The results show that serotonin significantly induced proliferation in metacestode vesicles and stimulated vesicle development in primary (stem) cell cultures. We also were able to demonstrate that inhibition of serotonin transport with the selective serotonin reuptake inhibitor (SSRI) paroxetine affected both the structural integrity of metacestode vesicles and the viability of primary parasite cells.

## Methods

### Parasite material

Parasite material was isolated from Mongolian jirds (*Meriones unguiculatus*) where it was maintained through serial peritoneal passages, as described previously [31, 33]. Parasite axenic (host cell-free) primary stem cell cultures were set up from* in vitro*-cultivated metacestode vesicles essentially as previously described [31, 33]. Protoscoleces were obtained from parasite material freshly isolated from Mongolian jirds, as described by Brehm et al. [34] with slight modifications. Briefly, parasite material in phosphate buffered saline (PBS) was shaken vigorously for 10 min to free protoscoleces from metacestode tissue to which they were attached. The suspension was first filtered through a 150-µm gauze to remove debris, then filtered through a 30-µm gauze filter that retained the protoscoleces. Protoscoleces were collected, resuspended in PBS and transferred to a Petri dish. Slow rotation of the dish separated out calcium bodies, which settled at the bottom, and the protoscoleces, which accumulated in the middle. Protoscoleces were then collected and activated by incubation in 0.05% w/v pepsin (pH 2 in Dulbecco's Modified Eagle Medium (DMEM) for 30 min at 37 °C and 125 rpm (modified from Fernández et al. [35]). After washing three times with PBS, protoscoleces were incubated in 0.2% w/v sodium taurocholate (pH 7.4 in DMEM) for 3 h at 37 °C and 125 rpm. Finally, protoscoleces were washed in PBS and fixed in 4% paraformaldehyde (w/v) overnight for use in whole mount* in situ* hybridizations (WMISH).

### Cloning and sequencing

For cloning and sequencing of the *E. multilocularis* serotonin transporter (SERT) cDNA (*sert*) and *E. multilocularis *tryptophan hydroxylase (TPH) cDNA (*tph*), primers were designed based on available genomic and predicted coding sequences (CDS) (EmuJ_000391300 and EmuJ_000069500, respectively; downloaded from GeneDB) [36–38]. Partially overlapping fragments were amplified from cDNA libraries [39] using different primer combinations (see Additional file 1: Table S1). Rapid amplification of cDNA ends (RACE) was performed for *E. multilocularis tph* using the plasmid primer IG4-5′ SPR2 (binding to the pJG4-5 plasmid) and an *E. multilocularis tph*-specific primer with cDNA libraries [39] as template. PCR products were cloned into pJet1.2 using the CloneJET^TM^ PCR Cloning Kit (Fermentas, St. Leon-Rot, Germany) and sequenced. Sequences of overlapping fragments were assembled in BioEdit 7.2.5 [40], and the assembled sequences were deposited at the EMBL Nucleotide Sequence Database under the accession numbers LT934126.1 (*E. multilocularis sert*) and LT934127.1 (*E. multilocularis tph*).

### RNA isolation and reverse transcription

RNA isolation was performed with the Direct-zol™ RNA MiniPrep kit (Zymo Research, Freiburg, Germany) according the manufacturer’s instructions (including DNase treatment). For reverse transcription, the SuperScript®IV Reverse Transcriptase (Invitrogen, Darmstadt, Germany) was used with an Oligo-dT primer (5′-ATC TCT TGA AAG GAT CCT GCA GGA CTT _22_VX-3′) according to the manufacturer’s instructions.

### Quantitative reverse transcription-PCR

Quantitative reverse transcription (RT-PCR) was performed on the StepOnePlus Real-Time PCR System (Thermo Fisher Scientific, Schwerte, Germany) using the following primers: 5′-CTC CTT CAA AGA GCG TTT G-3′ and 5′-TTC GGA CTG TGT ATC G-3′ for *E. multilocularis sert*; 5′-ATC AAC TCT GGA TGT GGT-3′ and 5′-GAG GTT AAA TGA TGC GGT GC-3′ for *E. multilocularis tph*; and 5′-TGA TGA AAG TGA AGC CAA GGA ACT TGA G-3′ and 5′-TTC GTC TGG AGC GTC TCA TTC TTA GAG-5′) for the reference gene *E. multilocularis elp*. The reaction mixture contained 2 μl of 1:5 diluted cDNA, 300 nM each primer (200 nM for *E. multilocularis sert*) and the HOT FIREPol®EvaGreen® qPCR Mix (ROX) (Solis Biodyne, Düsseldorf, Germany). The following cycling program was used: 95 °C, 15 min; then 95 °C/15 s, 60 °C/20 s (58 °C/20 s for *E. multilocularis sert*) for 40 cycles; and a final extension at 72 °C for 20 s; fluorescence was measured at 72 °C. The amplification product specificity was assessed by melting curve analysis. The experiment was carried out with three technical and three biological replicates. The amplification efficiency was calculated with linREG [41, 42], and the relative gene expression was calculated with the formula of Pfaffl [43]. Statistical analysis was performed in fgStatistics [44] using a permutation test with 5000 pairwise resampling cycles.

### Bioinformatic analyses

cDNA sequences for *E. multilocularis sert* (LT934126.1) and *E. multilocularis tph* (LT934127.1) were used for BLASTN searches (E-value < 1e−10, identities > 95%, coverage > 3%) against the *E. multilocularis* genome at WormBaseParaSite database (WBPS 9) [36, 45–47] to determine exon/intron boundaries. For domain analyses, cDNA sequences were translated into protein sequences with BioEdit 7.2.5 six-frame translations [40] which then were searched with SMART 8.0 for Pfam domains [48, 49]. To investigate conservation, BLASTP searches (E-value < 1e−10, identities > 50%, coverage > 90%) with the sequences of the active domains were performed against the non-redundant protein sequences of *Homo sapiens* (taxid: 9606) and *Schistosoma mansoni* (taxid: 6183) in the National Center for Biotechnology Information (NCBI) database. A multiple sequence alignment for the sodium neurotransmitter symporter family (SNF) domains of serotonin transporters from different organisms was generated in BioEdit 7.2.5 (40) using MUSCLE v3.8.31 (4 iterations) [50, 51].

### Transcriptome data analysis

Available RNA-Seq data [36] (ENA sample accessions: ERS094035, ERS094036, ERS094037, ERS094038, ERS094039, ERS016464, ERS018054, ERS018053) was mapped to the new version of the *E. multilocularis* genome downloaded from WormBaseParaSite WBPS7 [36, 45, 46, 52] using Hisat2 v2.0.5 [53]. Reads per transcript (annotations downloaded from WormBaseParaSite WBPS7) were counted with HTSeqCount v0.7.1 [54], using a minimum quality score of 30 to filter out low quality or multiple mapped reads. TPM (Transcripts Per kilobase of exon per Million transcripts mapped) values were calculated for all transcripts.

### Whole-mount *in situ* hybridization

For the synthesis of digoxigenin-labeled probes, cDNA fragments were amplified using the primers SERT_A_dw (5′-GAA TGC TGT AGA TGT GGT TAT GG-3′) and SERT2_up (5′-CTG GTC CCA CAG TTG ATT GC-3′) for *E. multilocularis sert*, and IG 4-5-SPR2 (5′-CTT ATG ATG TGC CAG ATT ATG-3′) and TPH_2_up (5′-CAG AGA GGC GAT ACC CAA CTC-3′) for *E. multilocularis tph*. PCR products were cloned into pJet1.2 using CloneJET^TM^ PCR Cloning Kit (Fermentas, St. Leon-Rot, Germany) and amplified with the primers T7 Plus 2 (5′-AGA AGA GTA ATA CGA CTC ACT ATA GG-3′) and 5-SP6+pJET1.2.Rev-3 (5′-ATA ATT TAG GTG ACA CTA TAG AAC ATC GAT TTT CCA TGG CAG-3′). Digoxigenin-labeled probes were synthesized by* in vitro* transcription with T7 and SP6 polymerases (New England Biolabs, Frankfurt/Main, Germany) according to the manufacturer’s instructions. WMISH for protoscoleces and metacestodes was performed as described previously [55] using the sense probe as control. Images of metacestodes and Z-stack images of protoscoleces were generated by confocal microscopy (Leica TCS SP5; Leica Microsystems, Wetzlar, Germany). All images were processed with Fiji/ImageJ 2.0.0 [56, 57]. A total of 18 protoscoleces from two different isolates (DPZ and MS1010) were analyzed for each gene. The micrographs shown in the Results section are Z-projections (maximum intensity) of 25 (*E. multilocularis sert*) and 19 (*E. multilocularis tph*) focal planes. The region of the protoscolex was cut out, converted to RGB values, made into a montage and flattened with the overlay.

### Chemicals

All compounds were purchased from Sigma-Aldrich (Munchen, Germany). The following stock solutions were prepared: 50 mM serotonin hydrochloride in 0.1 M hydrochloric acid; 50 mM paroxetine maleate salt in ethanol; and 4 mM 4-chloro-dl-phenylalanine in H_2_O. Solutions were filtrated through the Filtropur S 0.2 syringe filter system (Sarsteadt, Nuembrecht, Germany) and stored at − 20 °C prior to use.

### Serotonin treatment of primary cells

*Echinococcus multilocularis* primary cells were cultured in 96-well culture plates (Sarsteadt, Nuembrecht, Germany) with 200 µl conditioned medium (c-DMEM-A and c-DMEM-B 1:1, described in [33]) under a nitrogen atmosphere. Medium was supplemented with different concentrations of serotonin (1, 10 or 100 µM). The number of fully developed metacestode vesicles was determined by light microscopy after 2 weeks of cultivation. Three independent experiments with each three replicates were performed. The number of developed metacestode vesicles was normalized to the control of each experiment. Normalized data were statistically analyzed using GraphPad Prism 7.00 for Windows (GraphPad Software, La Jolla, CA, USA), using a Kruskal–Wallis test with a Dunn’s multiple comparisons test to compare all concentrations with the control.

### Labeling and detection of 5-ethynyl-2′-deoxyuridine

Metacestode vesicles with a diameter of 3–4 mm were cultured in a 12-well culture plate with 2 ml c-DMEM-A [33] supplemented with serotonin (1, 10 or 100 µM) for 7 days. Short-term labeling and subsequent whole-mount detection of the thymidine analogue 5-ethynyl-2′-deoxyuridine (EdU; Life Technologies, Darmstadt, Germany) were performed as described previously [55]. Samples were analyzed by confocal microscopy (Leica TCS SP5; Leica Microsystems, Wetzlar, Germany). For quantification of EdU-positive cells, two random fields per vesicle were captured, four vesicles per replicate, three replicates per concentration. Cells were identified based on DAPI (4′,6-diamidino-2-phenylindole) staining using a custom script in Fiji/ImageJ 2.0.0 [56, 57]. EdU-positive cells were counted manually. The mean percentage of EdU-positive cells was calculated for each replicate. Statistical analysis was performed with GraphPad Prism 7.00 for Windows (GraphPad Software, La Jolla, CA, USA) using an ordinary one-way analysis of variance (ANOVA) with a Dunnett’s multiple comparisons test comparing all concentrations with the control.

### Inhibitor treatment of metacestode vesicles

Metacestode vesicles were cultured in a 12-well-plate with 2 ml c-DMEM-A [33] supplemented with the SSRI paroxetine (1, 10 or 100 µM) or 4-chloro-dl-phenylalanine (1, 10 or 100 µM). The structural integrity of metacestode vesicles was determined by light microscopy and the number of damaged vesicles was counted. Experiments were performed with three biological replicates. The percentages of structurally affected metacestode vesicles were used for statistical analysis with GraphPad Prism 7.00 for Windows (GraphPad Software) using a two-way, repeated measurements (for the time factor) ANOVA with a Dunnett’s multiple comparisons test for each time point, comparing all concentrations with the control.

### Resazurin assay

Primary cells were cultured in 96-well plates (Sarsteadt, Nuembrecht, Germany), with 100 µl c-DMEM-A [33] supplemented with paroxetine (1, 10 or 100 µM) for 2 days. As cytotoxic control, 1% triton X-100 (Sigma-Aldrich) was used. A 100-µl aliquot of resazurin (Sigma-Aldrich) diluted in PBS was added to all wells (final concentration 10 µl/ml). After 3 h of incubation, fluorescence was measured at 540 nm (reference 595 nm) with a TECAN ELISA reader (Tecan Group, Crailsheim, Germany). Three independent experiments, each each three technical replicates, were carried out. Statistical analysis was performed with GraphPad Prism 7.00 for Windows (GraphPad Software) using an ordinary one-way ANOVA with a Dunnett’s multiple comparisons test to compare all samples with the negative control.

### Paroxetine treatment of primary cells for quantitative reverse transciption-PCR

Primary cells were cultured in 96-well plates (Sarsteadt) with 200 µl conditioned medium (c-DMEM-A and c-DMEM-B 1:1, described in [33]) under anaerobic conditions and treated with 10 µM paroxetine for 2 days. The cells were then resuspended and transferred to 1.5-ml tubes. After centrifugation at 600 *g* for 5 min, the supernatant was discarded and the cells were resuspended in 500 µM Trizol® Reagent (Invitrogen) and stored at − 80 °C until RNA isolation. The experiment was performed with three biological replicates.

## Results

### The *E. multilocularis* serotonin transporter and tryptophan hydroxylase show high homologies to their human homologues within the active domains

**Fig. 1:**
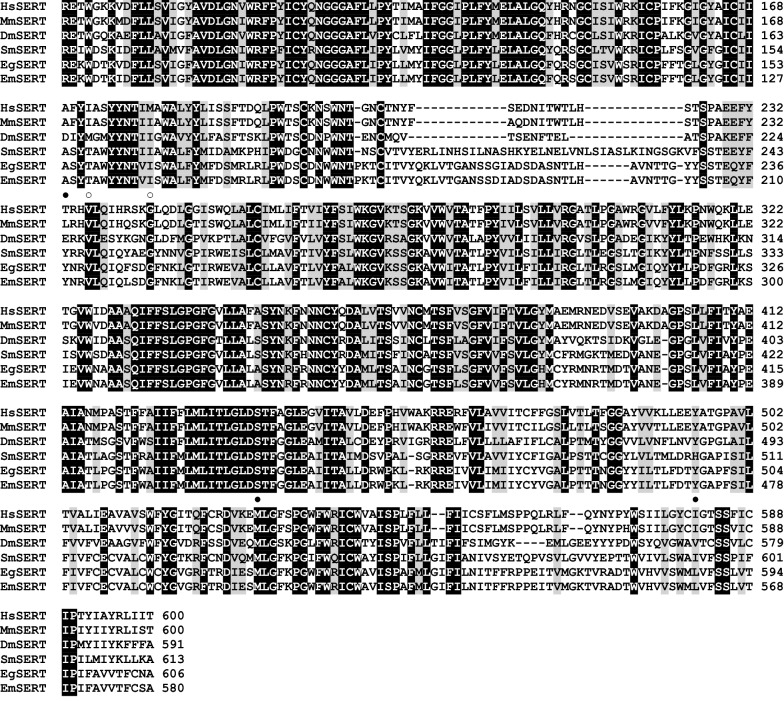
Comparison of the SNF domain of serotonin transporters (SERT). The multiple sequence alignment utility MUSCLE 3.8.31 [50, 51] was used to align amino acid sequences from *Homo sapiens* (*HsSERT*, NP_001036.1), *Mus musculus* (*MmSERT*, AAB67172.1), *Drosophila melanogaster* (*DmSERT*, NP_523846.2), *Schistosoma mansoni* (*SmSERT*, EF061308), *Echinococcus granulosus* (*EgSERT*, EUB59773.1) and *Echinococcus multilocularis* (*EmSERT*, LT934126.1) SERT. Highly conserved residues are printed white on black background, biochemically similar residues are printed black on gray background. Conserved binding sites for paroxetine [58–61] in *E. multilocularis* SERT are indicated by black circles; white ones identify substitutions

Previous* in silico* analyses showed that a canonical serotonergic pathway, with exception of the monoamine oxidase and serotonin receptor 3, is encoded in both the *E. granulosus* and *E. multilocularis* genomes. In the present study, we cloned and sequenced the *E. multilocularis* genes encoding the serotonin transporter (*E. multilocularis sert*: LT934126.1) and tryptophan hydroxylase (*E. multilocularis tph*: LT934127.1). *E. multilocularis sert* comprised 13 exons and 12 introns, localized on chromosome 9, and encoded a protein, *E. multilocularis* SERT, of 640 amino acids. Domain analysis with SMART 8.0 showed that *E. multilocularis* SERT contained a SNF domain (PF00209) with 12 transmembrane domains, demonstrating the typical domain structure of a neurotransmitter transporter with sodium symporter activity. The *E. multilocularis* SERT SNF domain showed high homologies to the *Schistosoma mansoni* (82% amino acid similarities) and the human (69% amino acid similarities) SERT SNF domains. Analysis of five paroxetine binding sites of mammalian SERTs [58–61] showed conservation of three residues in *E. multilocularis* SERT (see Fig. 1).

Using 5′-RACE-PCR we obtained a sequence for *E. multilocularis tph*. Transcriptome information [36] viewed with the Integrative Genomics Viewer [62, 63] suggests that the sequence did not represent the full transcript and was missing the 5′-end with the start codon (see Additional file 2: Figure S1). The partial sequence of *E. multilocularis tph* comprised 12 exons and 11 introns, localized on chromosome 3, and encoded a partial protein of 445 amino acids. SMART [48, 49] analysis revealed that the partial *E. multilocularis* TPH protein contained a complete biopterin-dependent aromatic amino acid hydroxylase (Biopterin_H) domain (PF00351) that is characteristic of TPH and other aromatic amino acid hydroxylases. Within the Biopterin_H domain, *E. multilocularis* TPH showed high homologies to the *S. mansoni* TPH (82% amino acid similarities) and to the human TPH2 (81% amino acid similarities), which is predominantly expressed in the brain stem [64].

### Expression pattern of* E. multilocularis sert* and *E. multilocularis tph *indicates expression in the nervous system of the protoscolex

**Fig. 2:**
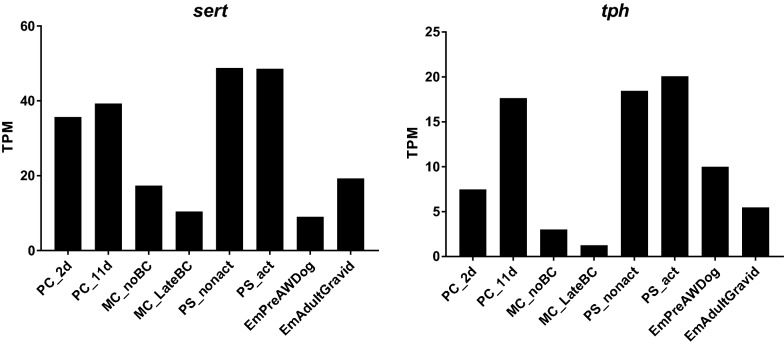
Gene expression profiles of *E. multilocularis sert* and *E. multilocularis tph*. Available transcriptome data [36] were mapped to the new genome version and gene expression was calculated. The units are transcripts per kilobase of exon per million transcripts mapped (*TPM*) values of *E. multilocularis sert* and *E. multilocularis tph* for primary cells (*PC_2d*: 2 days old,* PC_11d*: 11 days old), metacestode vesicles without (*MCnoBC*) and with (*MC_LateBC*) brood capsules, non-activated (*PS_nonact*) and activated protoscoleces (*PS_act*), pregravid (*EmPreAWDog*) and gravid adults (*EmAdultGravid*)

The expression of both *E. multilocularis sert* and *E. multilocularis tph* was analyzed in the available transcriptome data sets [36]. While *E. multilocularis sert* was generally more highly expressed than *E. multilocularis tph*, their expression profiles were similar. In comparison to the other analyzed life-cycle stages, both *E. multilocularis sert* and *tph* showed high expression in non-activated and activated protoscoleces and low expression in the metacestode (see Fig. 2).

It should be noted that a serotonergic nervous system has been described for protoscoleces of *E. multilocularis* and *E. granulosus* [4, 5, 13, 14], while no serotonergic nerve cells have been reported in in metacestode vesicles [4, 5].

**Fig. 3:**
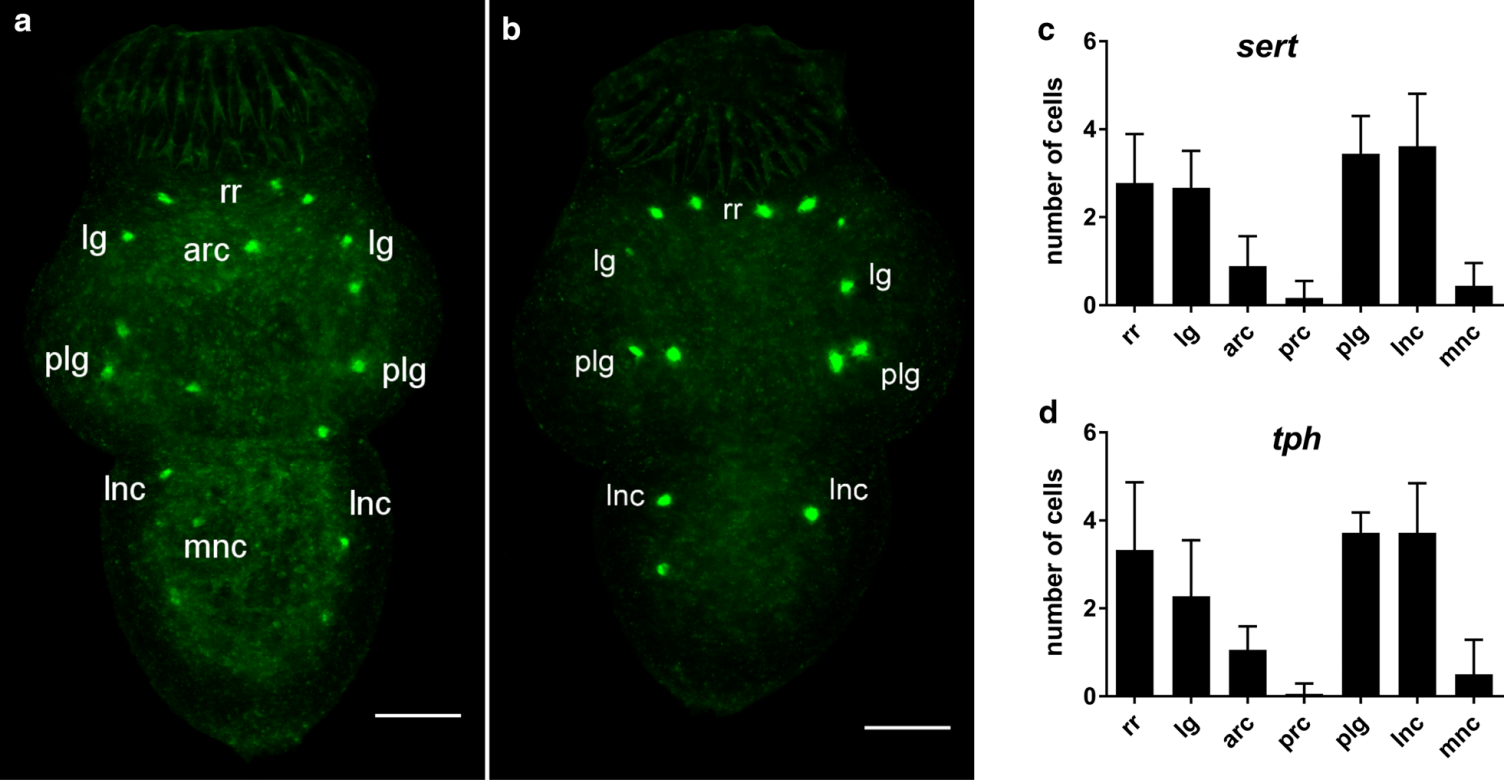
Expression of *E. multilocularis sert* and *E. multilocularis tph* in protoscoleces. **a** Whole mount* in situ* hybridization (WMISH) of *E. multilocularis sert*, **b** WMISH of *E. multilocularis tph,* **c** distribution of *E. multilocularis sert*-positive cells, **d** distribution of *E. multilocularis tph*-positive cells. *Echinococcus multilocularis sert*- and *E. multilocularis tph*-positive cells, respectively, were located in the rostellar ring (*rr*), the lateral ganglion (*lg*), the region of the anterior ring commissure (*arc*), the posterior lateral ganglion (*plg*), the lateral nerve cords (*lnc*), the medial nerve cords (*mnc*) and rarely in the posterior ring commissure (*prc*). **a**, **b** Micrographs are Z-projections (maximum intensity) of several focal planes. Bars: 25 µM. **c**, **d** Mean number of cells per protoscolex at each position (*n* = 18, isolates DPZ and MS1010). Error bars represent the standard deviation (SD)

To determine the expression patterns of *E. multilocularis sert* and *E. multilocularis tph* in protoscoleces, we performed WMISH using an established protocol [55]. Both the positions of *E. multilocularis sert*- and *E. multilocularis tph*-positive cells were relatively constant and corresponded to the location of the serotonergic nervous system in protoscoleces, as previously described [4, 5] (see Fig. 3). *Echinococcus multilocularis sert*- and *E. multilocularis tph*-positive cells, respectively, were positioned in the regions of the rostellar ring, the lateral ganglion, the posterior lateral ganglion and the lateral nerve cords. In a few cases, cells were also located in the region of the anterior ring commissure and the medial nerve cords. Rarely did we observe *E. multilocularis sert*- or *E. multilocularis tph*-positive cells in the region of the posterior ring commissure. In WMISH experiments for *E. multilocularis sert* or *tph* on metacestode vesicles, we did not obtain signals for either gene, which might be due to low expression in the metacestode stage.

Taken together, the WMISH and the transcriptome analysis suggest that *E. multilocularis sert* and *E. multilocularis tph* are expressed in the nervous system of the protoscolex.

### Serotonin stimulates *E. multilocularis* larval development* in vitro*

**Fig. 4:**
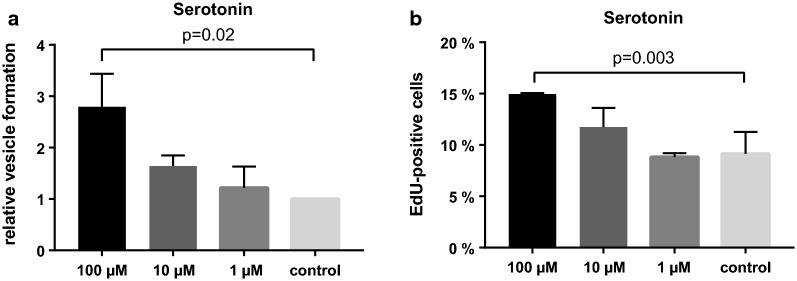
Effect of serotonin on *E. multilocularis*. **a** Metacestode vesicle development from primary cells in the presence of serotonin. Shown is relative vesicle formation compared to untreated control cultures. Error bars represent the SD. **b** Proliferation of metacestode vesicles treated with serotonin for 7 days. Shown are the percentages of thymidine analogue 5-ethynyl-2′-deoxyuridine (EdU)-positive cells. Error bars represent the SD

To study the influence of serotonin on parasite development, serotonin was applied exogenously to primary cell cultures and metacestode vesicles. Primary cells isolated from metacestode vesicles contain a high percentage of pluripotent stem cells [55] and, after the formation of aggregates, develop into metacestode vesicles [31]. This process mimicks the transition from the oncosphere to the metacestode during the early stage of parasite infection in the intermediate host [31, 65]. Exogenous serotonin stimulated the formation of metacestode vesicles from primary cells in a dose-dependent manner. After 14 days, significantly (*P* = 0.02) more metacestode vesicles were detected in samples treated with 100 µM serotonin compared to controls (see Fig. 4a). As an indicator for proliferation in metacestodes, we analyzed EdU incorporation and observed that the treatment of mature metacestode vesicles with serotonin stimulated the incorporation of EdU in a dose-dependent manner. Incubation with 100 µM serotonin for 7 days significantly (*P* = 0.003) increased the number of EdU-positive cells (see Fig. 4b), indicating proliferation of germinative cells.

In summary, exogenous serotonin stimulates EdU incorporation in metacestodes and vesicle formation in primary cells in *E. multilocularis*.

### Paroxetine affects parasite integrity and cell viability

**Fig. 5:**
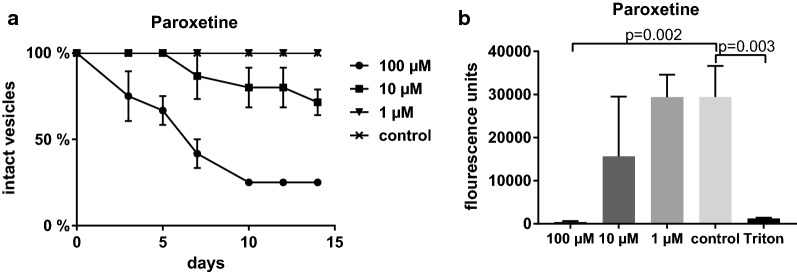
Effect of paroxetine on *E. multilocularis*. **a** Time course of structural integrity of metacestode vesicles in the presence of the selective serotonin reuptake inhibitor paroxetine. Shown are the percentages of intact vesicles. Error bars represent the SD. **b** Viability of primary cells after 2 days of treatment with the concentrations of paroxetine indicated. 1% triton X-100 was used as the cytotoxic control. Shown are fluorescence values. Error bars represent the SD

To further study the role of serotonin in *E. multilocularis*, we applied the SSRI paroxetine to parasite cultures. Paroxetine affected the structural integrity of metacestode vesicles. As early as 3 days after exposure, cultures incubated with 100 µM paroxetine contained significantly (*P* = 0.006) more collapsed vesicles than untreated control cultures. After 10 days also metacestode cultures treated with 10 µM paroxetine showed significantly (*P* = 0.03) more damaged vesicles than control cultures. In contrast, metacestodes incubated with 1 µM paroxetine and untreated metacestodes remained intact (see Fig. 5a). On day 14 of the experiment, cultures incubated with 10 µM paroxetine contained 29% collapsed metacestodes, with cultures treated with 100 µM paroxetine even reaching 75% collapsed metacestodes.

To investigate if paroxetine affects cell viability, we performed a resazurin assay on primary cells. Treatment with 100 µM paroxetine for 2 days strongly reduced cell viability compared to untreated controls (*P* = 0.002). Incubation with 10 µM paroxetine also affected cell viability, although to a lesser extent, and not in a statistically significant manner. Paroxetine at the concentration of 1 µM had no effect on primary cells.

To address the question if SERT inhibition by paroxetine is accompanied by a change in expression of *E. multilocularis sert* and* tph*, we performed quantitative RT-PCR on cDNA from primary cells. Treatment with 10 µM paroxetine for 2 days did not significantly alter the expression of *E. multilocularis sert* and* tph* (1.03 and 1.16 relative expression compared to untreated controls, respectively; see Additional file 3: Figure S2) suggesting that treatment with paroxetine has no or only a slight effect on the expression of *E. multilocularis sert* and* tph*.

Taken together, these results indicate that serotonin transport is crucial for the integrity of *E. multilocularis* metacestode vesicles and the viability of primary cells.

### 4-Chloro-dl-phenylalanine does not affect structural integrity of metacestodes

4-Chloro-dl-phenylalanine is a known inhibitor of TPH [66], which is the rate-limiting enzyme of serotonin synthesis [67]. To study the role of endogenous serotonin synthesis, we applied 4-chloro-dl-phenylalanine to parasite cultures. Even after 21 days of incubation with 4-chloro-dl-phenylalanine at different concentrations (100, 10 and 1 µM), metacestodes showed no signs of structural damage. Treatment with 4-chloro-dl-phenylalanine for 2 days also did not affect the viability of primary cells (see Additional file 4: Figure S3).

## Discussion

Serotonin is a phylogenetically ancient molecule with various functions. In addition to its role as a neurotransmitter, serotonin regulates many developmental processes across phyla [19, 22, 68]. In a previous work we and others showed that the *E. granulosus* genome encodes the proteins required for serotonin biosynthesis, transport and sensing, and that homologues are also present in the *E. multilocularis* genome [4]. In the present study, we sequenced the genes encoding the *E. multilocularis* SERT, which is required for the uptake of serotonin, and the *E. multilocularis* TPH, which is the rate-limiting enzyme in endogenous serotonin synthesis [67]. *Echinococcus multilocularis* TPH contains a Biopterin-dependent aromatic amino acid hydroxylase domain and shows high homologies to the *Schistosoma mansoni* and human TPH, indicating conservation of the enzyme in metazoa. Sequence analysis of *E. multilocularis* SERT revealed a SNF domain, which is a characteristic of sodium-dependent neurotransmitters, and high homologies to *S. mansoni* and human SERT. As not all binding sites for paroxetine are conserved in the *E. multilocularis* SERT and the closely related *S. mansoni* SERT has lower binding affinity for paroxetine than human SERT [69], we used high concentrations of paroxetine in our experiments. While off-target effects cannot be excluded, the binding properties of paroxetine together with the observed effects of paroxetine even at the lower concentration of 10 µM suggest that the detected effects are specific.

To investigate the localization of cells expressing *E. multilocularis sert* and *tph*, we performed WMISH on protoscoleces. The positions and frequencies of cells expressing *E. multilocularis sert* and *tph* corresponded to the positions and frequencies of serotonin-containing neurons, as described previously [5], suggesting that both genes are expressed in the nervous system of the protoscolex. A further method to verify this would be co-localization studies using a specific marker for the *E. multilocularis* nervous system; however, at the present time there are no methods to perform such a study. Similar to our findings, WMISH and immunofluorescence analysis of the planarian *Dugesia japonica* show expression of the TPH in the planarian nervous system [70]. In addition, available transcriptome data show high expression levels of *E. multilocularis sert* and *tph* in non-activated and activated protoscoleces, which could be attributed to expression in the nervous system of the protoscolex. In comparison, the expression of *E. multilocularis sert* and *tph* in adults is surprisingly low considering the complexity of the serotonergic nervous system described for adults of the closely related species *E. granulosus* [4, 14]. The relatively high expression of *E. multilocularis sert* and *tph* in primary cells could be explained by the presence of nerve cells in primary cell preparations [55]. Likewise, the absence of a serotonergic nervous system at the metacestode stage [5] could account for the low expression levels of *E. multilocularis sert* and *tph* in metacestodes. Also, we were not able to detect a specific signal in the WMISH of metacestodes for either gene, which could be due to expression in only a few cells or very low expression in many cells. Taken together, the expression profiles of *E. multilocularis sert* and *tph* indicate the expression of both genes in nerve cells.

As noted above, serotonin regulates many developmental processes across phyla [19, 22, 68]. Our results indicate that serotonin stimulates the development of *E. multilocularis* metacestode vesicles from primary cell preparations and induces proliferation in metacestodes. Serotonin could therefore be an important developmental signal during the formation and growth of the metacestode in the liver. Considering that basal serotonin levels in human blood plasma [20] and in various tissues [71] are lower than 100 nM, we observed developmental effects at quite high concentrations. However, serotonin concentrations can reach higher levels during inflammation [71]. Other possibilities are increased serotonin concentrations through endogenous serotonin synthesis or uptake of exogenous serotonin.

The authors of previous studies have proposed that cellular uptake is required for certain mitogenic and developmental effects of serotonin [20, 72, 73]. Serotonin transport is known to be involved in developmental processes in flatworms, such as during the traumatic regeneration of the planarian *Polycelis tenuis* [27] and the miracidial transformation of *S. mansoni* [28, 29]. We have previously reported that exogenous serotonin induces re-differentiation of *E. granulosus* protoscoleces towards the metacestode stage and that this process is inhibited by citalopram [4]. Here, we have shown that the SSRI paroxetine significantly reduced the viability of primary cells and affected the structural integrity of metacestode vesicles, indicating that serotonin transport is essential for the parasite. Since primary cell cultures are highly enriched in stem cells [55], we expect that paroxetine has not only toxic effects on primary cells but also on stem cells in metacestodes, which might account for the loss of structural integrity of paroxetine-treated metacestodes. While it cannot be ruled out that paroxetine treatment affects the expression of *E. multilocularis sert* and *tph*, we found no evidence in that regard, suggesting that the effects of paroxetine cannot be compensated for by the upregulation of serotonin transport or endogenous serotonin synthesis.

Metacestodes lack a serotonergic nervous system [5]. Since, according to transcriptome information reported here, *E. multilocularis sert* was expressed in metacestodes and the SSRI paroxetine showed clear effects on metacestodes, it is probable that *E. multilocularis* SERT is not exclusively expressed in nerve cells but also in other cell types. We therefore hypothesize that *E. multilocularis* SERT fulfills an additional and essential function outside the nervous system. This hypothesis is in accordance with previous results on the *S. mansoni* SERT where the authors propose a predominantly neuronal function for the SERT with a possible secondary role in exogenous serotonin uptake [74]. In addition to a potential role in serotonin uptake from the host, the *E. multilocularis* SERT might transport serotonin into cells where it could act as an intracellular regulator of cell activity, an evolutionary early role of serotonin [19]. In contrast to *E. multilocularis sert*, expression of the *E. multilocularis tph* appears to be limited to nerve cells. Neither transcriptome information, WMISH nor cell culture experiments provided any indication of a role outside the nervous system. While it cannot be ruled out that the lack of response to 4-chloro-dl-phenylalanine in cell culture experiments is due to 4-chloro-dl-phenylalanine not inhibiting *E. multilocularis* TPH, this could also be explained by a lack of (nerve) cells that express TPH.

## Conclusions

Serotonin is a widely distributed neurotransmitter and mitogen. Our study provides evidence for both functions in *E. multilocularis*. Transcriptome data and WMISH suggest that the *E. multilocularis tph* and *sert* are expressed in the nervous system of the protoscolex. While the role of the *E. multilocularis* TPH appears to be restricted to the nervous system, our data indicate that the *E. multilocularis* SERT has an additional role outside the nervous system and is essential for parasite development and survival. Our data further suggest that serotonin plays an important role in *E. multilocularis* metacestode development and proliferation. Serotonin could therefore be a contributing factor to the formation and growth of the parasite in the liver.

## Supplementary Information

**Additional file 1: Table S1.** Primer combinations used for amplification of *E. multilocularis sert* and *E. multilocularis tph*.

**Additional file 2: Figure S1.** Genomic location of *E. multilocularis tph*.

**Additional file 3: Figure S2.** Effect of paroxetine on expression of *E. multilocularis sert* and *tph*.

**Additional file 4: Figure S3.** Effect of 4-chloro-dl-phenylalanine on *E. multilocularis*.

## Data Availability

The sequences of *E. multilocularis sert* and *tph* are available in the EMBL Nucleotide Sequence Database under the accession numbers LT934126.1 (*E. multilocularis sert*) and LT934127.1 (*E. multilocularis tph*), https://www.ebi.ac.uk/.

## Abbreviations

Biopterin_H: Biopterin-dependent aromatic amino acid hydroxylase
EdU: 5-Ethynyl-2′-deoxyuridine
RACE: Rapid amplification of cDNA ends
RT-PCR: Reverse transcription PCR
SERT: Serotonin reuptake transporter
SNF: Sodium neurotransmitter symporter family
SSRI: Selective serotonin reuptake inhibitor
TPH: Tryptophan hydroxylase
WMISH: Whole mount* in situ* hybridization
