# Supplementary material for: Serotonin stimulates Echinococcus multilocularis larval development
Source: Parasit Vectors. 2021 Jan 6;14:14. doi: 10.1186/s13071-020-04533-0 (PMC7789706; doi:10.1186/s13071-020-04533-0)
Supplement: Supplementary file 1 — Additional file 1: Table S1. Primer combinations used for amplification of E. multilocularis sert and E. multilocularis tph. [file 13071_2020_4533_MOESM1_ESM.pdf]

Fragment description	Downstream primer	Upstream primer
SERT A-2	SERT_A_dw (5'-GAA TGC TGT AGA TGT GGT TAT GG-3')	SERT2_up (5'-CTG GTC CCA CAG TTG ATT GC-3')
SERT 1-2	SERT1_dw (5'-GAT GCC GTT GTG GTG GAG AC-3')	SERT2_up (5'-CTG GTC CCA CAG TTG ATT GC-3')
SERT 2-Z	SERT2_dw (5'-GCA ATC AAC TGT GGG ACC AG-3')	SERT_Z_up (5'-GAT TGG TGC AAT GGG GAG-3')
TPH 2-3	TPH_2_dw (5'-GAG TTG GGT ATC GCC TCT CTG-3')	TPH_3_up (5'-GGA TCC GAG GGC TTG ACG-3')
TPH 1-2	TPH_1_dw (5'-GAC GCT GGT GAT GTC GTA ATT C-3')	TPH_2_up (5'-CAG AGA GGC GAT ACC CAA CTC-3')
TPH 2-Z	TPH_2_dw (5'-GAG TTG GGT ATC GCC TCT CTG-3')	TPH_Z_up (5'- GAG GTT AAA TGA TGC GGT GC-3')
TPH 5' RACE - 2	IG 4-5-SPR2 (5'-CTT ATG ATG TGC CAG ATT ATG-3')	TPH_2_up (5'-CAG AGA GGC GAT ACC CAA CTC-3')

Table S1 Primer combinations used for amplification of *E. multilocularis sert* and *E. multilocularis tph.*
