# Supplementary material for: Serotonin stimulates Echinococcus multilocularis larval development
Source: Parasit Vectors. 2021 Jan 6;14:14. doi: 10.1186/s13071-020-04533-0 (PMC7789706; doi:10.1186/s13071-020-04533-0)
Supplement: Supplementary file 2 — Additional file 2: Figure S1. Genomic location of E. multilocularis tph. [file 13071_2020_4533_MOESM2_ESM.pdf]

```
                             12719310  12719320  12719330  12719340  12719350  12719360  12719370
                           ....|....|....|....|....|....|....|....|....|....|....|....|....|....|
>pathogen_EmW_scaffold_03  CCATTCGGTGACATAAAACTGGGAAATGCTTGCAAAGGAATCACCTCACCTAGCACTGGTCGTGGGAAAG
 >LT934127.1               -------------------------------------GAATCACCTCACCTAGCACTGGTCGTGGGAAAG
                                                     M  L  A  K  E  S  P  H  L  A  L  V  V  G  K

                             12719380  12719390  12719400  12719410  12719420  12719430  12719440
                           ....|....|....|....|....|....|....|....|....|....|....|....|....|....|
>pathogen_EmW_scaffold_03  GATGCCAAAGTGAAGGAATTCGCAGCACTTCTTAGAAGATTGCAGGTACTAGCCATTCTTTTTATTATA…
>LT934127.1                GATGCCAAAGTGAAGGAATTCGCAGCACTTCTTAGAAGATTGCAG-------------------------
                            D  A  K  V  K  E  F  A  A  L  L  R  R  L  Q

                             12720220  12720230  12720240  12720250  12720260  12720270  12720280
                           ....|....|....|....|....|....|....|....|....|....|....|....|....|....|
>pathogen_EmW_scaffold_03 …CAAATTTTAGGAAATAAATCATGGCATTGACGGTATCAGGGTCGTGGACGCTGGTGATGTCGTAATTCAC
>LT934127.1                ----------GAAATAAATCATGGCATTGACGGTGTCAGGGTCGTGGACGCTGGTGATGTCGTAATTCAC
                                      E  I  N  H  G  I  D  G  V  R  V  V  D  A  G  D  V  V  I  H

                             12720290  12720300  12720310  12720320  12720330  12720340  12720350
                           ....|....|....|....|....|....|....|....|....|....|....|....|....|....|
>pathogen_EmW_scaffold_03  TCAAAATCTTCTGCTACTTTTAAGGATGACGTAATTCAAACTCTCGAAATCGTCTGCAAGGGCGTGACTG
>LT934127.1                TCAAAATCTTCTGCTACTTTTAAGGATGACGTAATTCAAACTCTCGAAATCGTCTGCAAGGGCGTGACTG
                            S  K  S  S  A  T  F  K  D  D  V  I  Q  T  L  E  I  V  C  K  G  V  T

                             12720360  12720370  12720380  12720390  12720400  12720410  12720420
                           ....|....|....|....|....|....|....|....|....|....|....|....|....|....|
>pathogen_EmW_scaffold_03  TAAAAACTGTGGATAACTTAAAAGCCAGTCTATCCATTCCCATTTTTGAAGGTTTGCAAACTGCTACCAC…
>LT934127.1                TAAAAACTGTGGATAACTTAAAAGCCAGTCTATCCATTCCCATTTTTGAAG-------------------
                           V  K  T  V  D  N  L  K  A  S  L  S  I  P  I  F  E

                             12720710  12720720  12720730  12720740  12720750  12720760  12720770
                           ....|....|....|....|....|....|....|....|....|....|....|....|....|....|
>pathogen_EmW_scaffold_03 …AGTAAAATTTTTGTTTTTAATTATCGTCAGAAAGCACAGTGGTGCCCTGGTTTCCACGACACATTTCTGA
>LT934127.1                ------------------------------AAAGCACAGTGGTGCCCTGGTTTCCACGACACATTTCTGA
                                                         E  S  T  V  V  P  W  F  P  R  H  I  S  E

                             12720780  12720790  12720800  12720810  12720820  12720830  12720840
                           ....|....|....|....|....|....|....|....|....|....|....|....|....|....|
>pathogen_EmW_scaffold_03  ACTGGACGCGGTCTCCAACAACGTCTTTATGTATGGAAAGGATCTGGATGCTGACCACCCTAGTTTTAAG
>LT934127.1                ACTGGACGCGGTCTCCAACAACGTCTTTATGTATGGAAAGGATCTGGATGCTGACCACCCTAGTTTTAAG
                             L  D  A  V  S  N  N  V  F  M  Y  G  K  D  L  D  A  D  H  P  S  F  K

                             12720850  12720860  12720870  12720880  12720890  12720900  12720910
                           ....|....|....|....|....|....|....|....|....|....|....|....|....|....|
>pathogen_EmW_scaffold_03  GATGAGGCATATAGAAAACGGAGAATGGAATTCGCCAAAATTGCTTACAATTACCGCTAGTGAGTTACAA…
>LT934127.1                GATGAGGCATATAGAAAACGGAGAATGGAATTCGCCAAAATTGCTTACAATTACCGCTA-----------
                            D  E  A  Y  R  K  R  R  M  E  F  A  K  I  A  Y  N  Y  R  Y

                             12721410  12721420  12721430  12721440  12721450  12721460  12721470
                           ....|....|....|....|....|....|....|....|....|....|....|....|....|....|
>pathogen_EmW_scaffold_03 …TAATCATCTTTTGTGATTTTTTAAATAAGTGGCGATGCAATTCCCCGTATTGAGTACACAAAGGAAGAAC
>LT934127.1                -----------------------------TGGCGATGCAATTCCCCGTATTGAGTACACAAAGGAAGAAC
                                                         G  D  A  I  P  R  I  E  Y  T  K  E  E

                             12721480  12721490  12721500  12721510  12721520  12721530  12721540
                           ....|....|....|....|....|....|....|....|....|....|....|....|....|....|
>pathogen_EmW_scaffold_03  GGAAGACGTGGTGAGTGTGACCTGACAGCATTTGCAAATGTCGCCTTCATCAGTAGCCCTGATGAGTCAG
>LT934127.1                GGAAGACGTG------------------------------------------------------------
                           R  K  T  W

                             12721550  12721560  12721570  12721580  12721590  12721600  12721610
                           ....|....|....|....|....|....|....|....|....|....|....|....|....|....|
>pathogen_EmW_scaffold_03  GCCAAAATGTCCTCTTCCTTCTTTATTTTTCATTCTCACAGGGGTATAGTTTATCGTGCATTAATGGAAC
>LT934127.1                -----------------------------------------GGGTATAGTTTATCGTGCATTAATGGAAC
                                                                     G  I  V  Y  R  A  L  M  E

                             12721620  12721630  12721640  12721650  12721660  12721670  12721680
                           ....|....|....|....|....|....|....|....|....|....|....|....|....|....|
>pathogen_EmW_scaffold_03  TCTATCGTACTCACGCCTGTAAGGAGTACTTGGAGAACATTCCCCTTCTTCAGGAACACGCAGGCTATCG
>LT934127.1                TCTATCGTACTCACGCCTGTAAGGAGTACTTGGAGAACATTCCCCTTCTTCAGGAACACGCAGGCTATCG
                           L  Y  R  T  H  A  C  K  E  Y  L  E  N  I  P  L  L  Q  E  H  A  G  Y  R

                             12721690  12721700  12721710  12721720  12721730  12721740  12721750
                           ....|....|....|....|....|....|....|....|....|....|....|....|....|....|
>pathogen_EmW_scaffold_03  GTATGCCCCACCACTGCTCCCTTATCATCACCGCTTTTGTGTCTAGGGAGGAGGATTTGCCCCAATTGGA
>LT934127.1                ----------------------------------------------GGAGGAGGATTTGCCCCAATTGGA
                                                                          E  E  D  L  P  Q  L  E
```

```
                                12721760  12721770  12721780  12721790  12721800  12721810  12721820
                           ....|....|....|....|....|....|....|....|....|....|....|....|....|....|
>pathogen_EmW_scaffold_03  GGATGTCTCCAACTTTCTCAAAAGTAAGCCTGTGGGGTTCGCTAATGTTTCGTGTATTAACTTTCTCCCT
>LT934127.1                GGATGTCTCCAACTTTCTCAAAA-----------------------------------------------
                             D  V  S  N  F  L  K

                                12721830  12721840  12721850  12721860  12721870  12721880  12721890
                           ....|....|....|....|....|....|....|....|....|....|....|....|....|....|
>pathogen_EmW_scaffold_03  TTTTTTGCATAGAGCAGTCGGGATTTACATTGCGTCCGGTTGCGGGCTACATTTCTGCTCGAGACTTTCT
>LT934127.1                ------------AGCAGTCGGGATTTACATTGCGTCCGGTTGCGGGCTACATTTCTGCTCGAGACTTTCT
                                       K  Q  S  G  F  T  L  R  P  V  A  G  Y  I  S  A  R  D  F  L

                                12721900  12721910  12721920  12721930  12721940  12721950  12721960
                           ....|....|....|....|....|....|....|....|....|....|....|....|....|....|
>pathogen_EmW_scaffold_03  TCAAGGCTTGGCTTATCGTGTATTCTACTGCACTCAGTACATTCGTCATAAAGCCGACCCCTTCTATACC
>LT934127.1                TCAAGGCTTGGCTTATCGTGTATTCTACTGCACTCAGTACATTCGTCATAAAGCCGACCCCTTCTATACC
                             Q  G  L  A  Y  R  V  F  Y  C  T  Q  Y  I  R  H  K  A  D  P  F  Y  T

                                12721970  12721980  12721990  12722000  12722010  12722020  12722030
                           ....|....|....|....|....|....|....|....|....|....|....|....|....|....|
>pathogen_EmW_scaffold_03  CCTGAACCGTAAGTAGCTTTAGCAGATCCACACAGAAGGCGTGAGGATCTAGTGGCGGAGATGGCGTGAG…
>LT934127.1                CCTGAACC--------------------------------------------------------------
                            P  E  P

                                12722390  12722400  12722410  12722420  12722430  12722440  12722450
                           ....|....|....|....|....|....|....|....|....|....|....|....|....|....|
>pathogen_EmW_scaffold_03 …TTCCCGATTAGGGATTGCATTCACGAATTGATGGGGCACGTACCACTACTGGCAAATCCGGAGTTTGCCC
>LT934127.1                -----------GGATTGCATTCACGAATTGATGGGGCACGTACCACTACTGGCAAATCCGGAGTTTGCCC
                                        D  C  I  H  E  L  M  G  H  V  P  L  L  A  N  P  E  F  A

                                12722460  12722470  12722480  12722490  12722500  12722510  12722520
                           ....|....|....|....|....|....|....|....|....|....|....|....|....|....|
>pathogen_EmW_scaffold_03  AGTACTCCCAGGAGTTGGGTATCGCCTCTCTGGGTGTCTCTGAAGAAGACGTTCAAAAACTAGCCAAGGT…
>LT934127.1                AGTACTCCCAGGAGTTGGGTATCGCCTCTCTGGGTGTCTCTGAAGAAGACGTTCAAAAACTAGCCAAG--
                           Q  Y  S  Q  E  L  G  I  A  S  L  G  V  S  E  E  D  V  Q  K  L  A  K

                                12723090  12723100  12723110  12723120  12723130  12723140  12723150
                           ....|....|....|....|....|....|....|....|....|....|....|....|....|....|
>pathogen_EmW_scaffold_03 …AGCTCTACTTCTTCTCTGTGGAATTTGGAATCTGCAATCAGGACGGAGAACTGCGCGCCTTCGGGGCTGG
>LT934127.1                --CTCTACTTCTTCTCTGTGGAATTTGGAATCTGCAATCAGGACGGAGAACTGCGCGCCTTCGGGGCTGG
                              L  Y  F  F  S  V  E  F  G  I  C  N  Q  D  G  E  L  R  A  F  G  A  G

                                12723160  12723170  12723180  12723190  12723200  12723210  12723220
                           ....|....|....|....|....|....|....|....|....|....|....|....|....|....|
>pathogen_EmW_scaffold_03  ACTGCTGTCTTCTGTGGCTGAATTAAAGGTGAGACCGCTGCAAATGCCGAAAAGTTGTAGGATATAAGAG…
>LT934127.1                ACTGCTGTCTTCTGTGGCTGAATTAAAG------------------------------------------
                             L  L  S  S  V  A  E  L  K

                                12723790  12723800  12723810  12723820  12723830  12723840  12723850
                           ....|....|....|....|....|....|....|....|....|....|....|....|....|....|
>pathogen_EmW_scaffold_03 …TGCTAGCTCTGAGGCACTTCTTCCCCTTCCAACTCCCAGCACGCATTAAGCGATGCCGCGGTGAGGATGC
>LT934127.1                ---------------------------------------CACGCATTAAGCGATGCCGCGGTGAGGATGC
                                                                   H  A  L  S  D  A  A  V  R  M

                                12723860  12723870  12723880  12723890  12723900  12723910  12723920
                           ....|....|....|....|....|....|....|....|....|....|....|....|....|....|
>pathogen_EmW_scaffold_03  CATTCATCCCCGAAGAGGTCATCAAGGCGGAATGCATGGTGACAACCTATCAAAAGGCTTACTTCACAAC
>LT934127.1                CATTCATCCCCGAAGAGGTCATCAAGGCGGAATGCATGGTGACAACCTATCAAAAGGCTTACTTCACAAC
                           P  F  I  P  E  E  V  I  K  A  E  C  M  V  T  T  Y  Q  K  A  Y  F  T  T

                                12723930  12723940  12723950  12723960  12723970  12723980  12723990
                           ....|....|....|....|....|....|....|....|....|....|....|....|....|....|
>pathogen_EmW_scaffold_03  CAGTACATTCAAAGAGGCACAAGACAAATTAAGGTCAGTCACATTTCTGGACGAAAAAGTTGAGTGTGTT…
>LT934127.1                CAGTACATTCAAAGAGGCACAAGACAAATTAAG-------------------------------------
                             S  T  F  K  E  A  Q  D  K  L  R

                                12724000  12724010  12724020  12724030  12724040  12724050  12724060
                           ....|....|....|....|....|....|....|....|....|....|....|....|....|....|
>pathogen_EmW_scaffold_03 …GTGCACAGTCTTCGAGTACCGCTATGCAAAATACGCGTTTCAGACACTTTGTTCAAAGCCTTCGTAGACC
>LT934127.1                -------------------------------------------ACACTTTGTTCAAAGCCTTCGTAGACC
                                                                        H  F  V  Q  S  L  R  R  P

                                12724070  12724080  12724090  12724100  12724110  12724120  12724130
                           ....|....|....|....|....|....|....|....|....|....|....|....|....|....|
>pathogen_EmW_scaffold_03  GTTTGCCCTTCGATACAATGCCTTTTCGGAGACTGTGGAGGTTTTGGATGAACCAAGAAAGCTTGGAATG
>LT934127.1                GTTTGCCCTTCGATACAATGCCTTTTCGGAGACTGTGGAGGTTTTGGATGAACCAAGAAAGCTTGGAATG
                             F  A  L  R  Y  N  A  F  S  E  T  V  E  V  L  D  E  P  R  K  L  G  M
```

```
                                   12724140  12724150  12724160  12724170  12724180  12724190  12724200
                             ....|....|....|....|....|....|....|....|....|....|....|....|....|....|
>pathogen_EmW_scaffold_03    CTATTAAAAGAAATGAAGGCTCAAATATCAACTCTGGAGGATGTGGTGGATCAGGTATGGTAATCTCCTC…
>LT934127.1                  CTATTAAAAGAAATGAAGGCTCAAATATCAACTCTGGAGGATGTGGTGGATCAG----------------
                              L  L  K  E  M  K  A  Q  I  S  T  L  E  D  V  V  D  Q

                                   12724350  12724360  12724370  12724380  12724390  12724400  12724410
                             ....|....|....|....|....|....|....|....|....|....|....|....|....|....|
>pathogen_EmW_scaffold_03   …GGCAGCACAATTTTCATTTTCTTTAGTTCTCCGACCGTCAAGCCCTCGGATCCACGGCACCGTGGAGGAT
>LT934127.1                  --------------------------TTCTCCGACCGTCAAGCCCTCGGATCCACGGCACCGTGGAGGAT
                                                        F  S  D  R  Q  A  L  G  S  T  A  P  W  R  M

                                   12724420  12724430  12724440  12724450  12724460  12724470  12724480
                             ....|....|....|....|....|....|....|....|....|....|....|....|....|....|
>pathogen_EmW_scaffold_03    GGCAGAGCCAAAAAAGTTCACTTAAAATGCACCGCATCATTTAACCTCTAGAGAACATAATTTTATGTTG
>LT934127.1                  GGCAGAGCCAAAAAAGTTCACTTAAAATGCACCGCATCATTTAACCTC----------------------
                               A  E  P  K  K  F  T  *
```

Figure S1 Genomic location of *E. multilocularis tph.* MUSCLE 3.8.31 (46, 47) alignment of the obtained cDNA sequence of *E. multilocularis tph* (LT934127.1) and the corresponding genomic region. Genomic positions are indicated above the alignment. The putative start codon is shown on grey background in the genomic sequence. The putative protein sequence for *E. multilocularis* TPH is shown below the alignment and underlined where supported by the obtained cDNA sequence.
