# Supplementary material for: Serotonin stimulates Echinococcus multilocularis larval development
Source: Parasit Vectors. 2021 Jan 6;14:14. doi: 10.1186/s13071-020-04533-0 (PMC7789706; doi:10.1186/s13071-020-04533-0)
Supplement: Supplementary file 3 — Additional file 3: Figure S2. Effect of paroxetine on expression of E. multilocularis sert and tph. [file 13071_2020_4533_MOESM3_ESM.pdf]

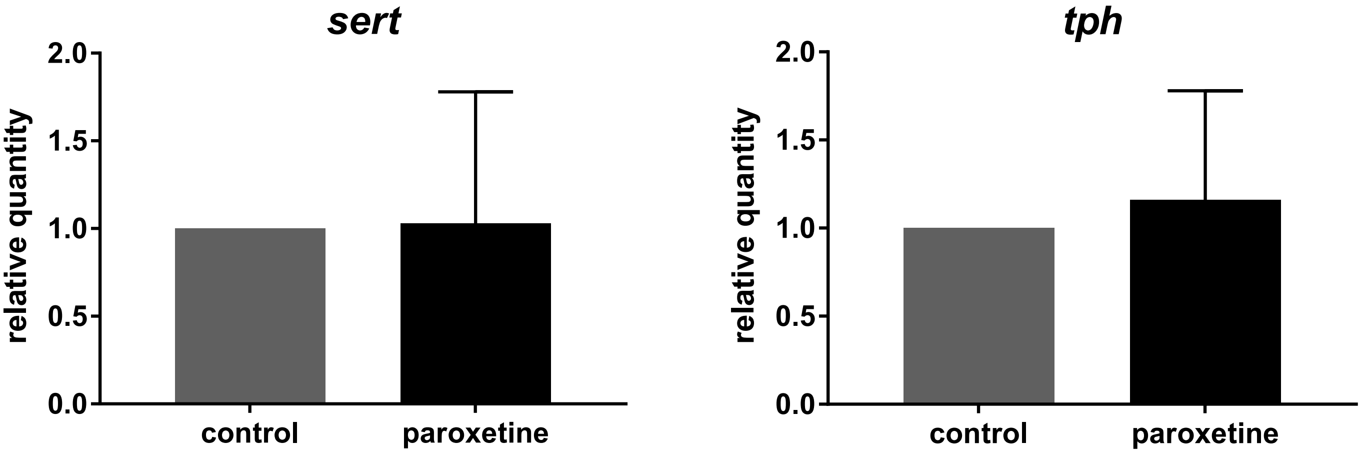

Figure S2 Effect of paroxetine on expression of *E. multilocularis sert* and *tph*. Expression of *E. multilocularis sert* and *tph* in primary cells treated with 10 µM paroxetine for 2 days compared to untreated controls. Shown are mean relative gene expressions. Error bars represent SE.
