# Supplementary material for: Serotonin stimulates Echinococcus multilocularis larval development
Source: Parasit Vectors. 2021 Jan 6;14:14. doi: 10.1186/s13071-020-04533-0 (PMC7789706; doi:10.1186/s13071-020-04533-0)
Supplement: Supplementary file 4 — Additional file 4: Figure S3. Effect of 4-chloro-dl-phenylalanine on E. multilocularis. [file 13071_2020_4533_MOESM4_ESM.pdf]

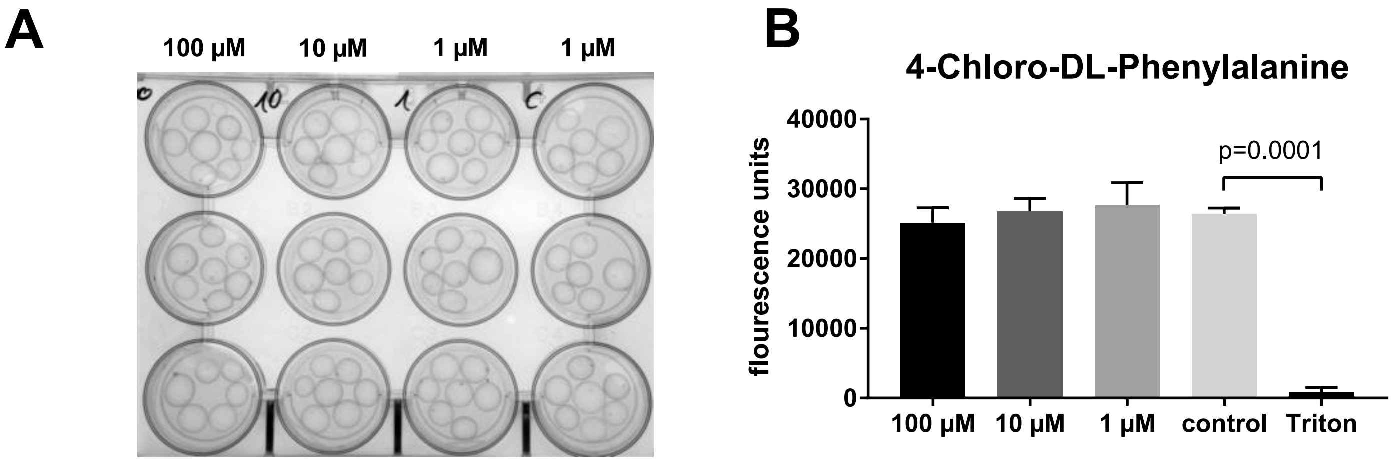

Figure S3 Effect of 4-chloro-DL-phenylalanine on *E. multilocularis*. A. Image of metacestode vesicles after 21 days of treatment with indicated concentrations of 4-chloro-DL-phenylalanine. **B.** Viability of primary cells after 2 day treatment with indicated concentrations of 4-chloro-DL-phenylalanine. 1 % triton X-100 was used as cytotoxic control. Shown are fluorescence values. Error bars represent SD.
